# H_2_O_2_ Signaling-Triggered PI3K Mediates Mitochondrial Protection to Participate in Early Cardioprotection by Exercise Preconditioning

**DOI:** 10.1155/2018/1916841

**Published:** 2018-07-25

**Authors:** Yang Yuan, Shan-Shan Pan, Dong-Feng Wan, Jiao Lu, Yue Huang

**Affiliations:** School of Kinesiology, Shanghai University of Sport, Shanghai, China

## Abstract

Previous studies have shown that early exercise preconditioning (EEP) imparts a protective effect on acute cardiovascular stress. However, how mitophagy participates in exercise preconditioning- (EP-) induced cardioprotection remains unclear. EEP may involve mitochondrial protection, which presumably crosstalks with predominant H_2_O_2_ oxidative stress. Our EEP protocol involves four periods of 10 min running with 10 min recovery intervals. We added a period of exhaustive running and a pretreatment using phosphoinositide 3-kinase (PI3K)/autophagy inhibitor wortmannin to test this protective effect. By using transmission electron microscopy (TEM), laser scanning confocal microscopy, and other molecular biotechnology methods, we detected related markers and specifically analyzed the relationship between mitophagic proteins and mitochondrial translocation. We determined that exhaustive exercise associated with various elevated injuries targeted the myocardium, oxidative stress, hypoxia-ischemia, and mitochondrial ultrastructure. However, exhaustion induced limited mitochondrial protection through a H_2_O_2_-independent manner to inhibit voltage-dependent anion channel isoform 1 (VDAC1) instead of mitophagy. EEP was apparently safe to the heart. In EEP-induced cardioprotection, EEP provided suppression to exhaustive exercise (EE) injuries by translocating Bnip3 to the mitochondria by recruiting the autophagosome protein LC3 to induce mitophagy, which is potentially triggered by H_2_O_2_ and influenced by Beclin1-dependent autophagy. Pretreatment with the wortmannin further attenuated these effects induced by EEP and resulted in the expression of proapoptotic phenotypes such as oxidative injury, elevated Beclin1/Bcl-2 ratio, cytochrome c leakage, mitochondrial dynamin-1-like protein (Drp-1) expression, and VDAC1 dephosphorylation. These observations suggest that H_2_O_2_ generation regulates mitochondrial protection in EEP-induced cardioprotection.

## 1. Introduction

Exercise is an intense stimulus factor that significantly enhances myocardial oxygen consumption, thereby resulting in myocardial hypoxia [1]. Repeated short-term exercise can cause recurrent transient absolute or relative myocardial ischemia, which is similar to the process of ischemic preconditioning (IP). Studies have shown that single-bout, high-intensity, intermittent aerobic exercise can induce endogenous cardioprotection in organisms, thereby protecting the myocardium during subsequent sustained ischemia [2]. This exercise-induced method of endogenous myocardial protection is known as exercise preconditioning (EP), which allows the heart to elicit adaptive responses to exhaustive exercise, thereby facilitating collateral myocardial damage [3, 4].

Acute cardiovascular stresses such as myocardial infarction (MI), ischemic reperfusion (I/R), or prolonged high-intensity exercise are strongly associated with a rapid increase in oxidative stress levels and morphological alterations in the mitochondria [5–11]. Therefore, oxidative stress acts as both inducer and reflector of mitochondrial dysfunction [12]. The majority of reactive oxygen species (ROS) (90%) induces oxidative stress, originates from the respiratory chain, and further generates hydrogen peroxide (H_2_O_2_) through the ROS scavenging effect of superoxide dismutase (SOD) [13]. The O_2_·− molecules do not readily pass through the mitochondrial membrane [14]. In this case, H_2_O_2_, which is mainly generated by the mitochondrial manganese-dependent superoxide dismutase (MnSOD), plays an important role in intracellular ROS signaling and the ROS-dependent mitophagy [15, 16]. Huang et al. [17] have shown that IP-induced mitophagy plays a cardioprotective role against acute ischemic injury. However, the association of exercise-induced cardioprotection to the activation of mitophagy remains unclear. We hypothesize that EEP-induced mitophagy, which is possibly triggered by H_2_O_2_ signaling, imparts cardioprotective effects.

Bcl-2/adenovirus E1B 19 kDa protein-interacting protein 3 (Bnip3) is a critical mitophagy receptor that is involved in the recruitment of autophagosome membrane protein microtubule-associated proteins 1 light chain 3 (MAP1LC3, also known as LC3) to the outer mitochondrial membrane (OMM), thereby inducing mitophagy and ultimately resulting in cardioprotection [18]. The subunit TOM20 is an important component of the translocase of the outer membrane (TOM) complex that indirectly contributes to mitophagy and participates in the mitochondrial repair in the heart [17, 19]. Through the direct coupling of autophagosomes to the mitochondria, the OMM-translocalized Bnip3, which has a C-terminal transmembrane domain, binds to LC3 via its LC3-interacting region (LIR) and acts as a proapoptotic protein through its BH-3-only domain [20]. A previous study has shown that Bnip3 responds to hypoxia and is thus strongly associated with oxidative stress [21]. In addition, Bnip3 activates other BH-3 proteins such as Bax/Bak and Beclin1, thereby resulting in their release from binding of the antiapoptosis protein Bcl-2 [22, 23]. Therefore, Bnip3 plays multiple roles, including OMM-pore opening, mitophagy mediation to the mitochondria, autophagic induction, and regulation of apoptosis in the cytosol. Furthermore, BH-3 proteins may be involved in mitochondrial fission [20, 24]. It should be an apparent cardioprotection if EEP regulates Bnip3-mediated mitophagy, yet requiring analysis of proapoptotic factors such as cytochrome c (Cyt-c) release and Beclin1/Bcl-2 ratio [25, 26].

Cellular phosphoinositide 3-kinase- (PI3K-) dependent macroautophagy plays an essential role in mitophagy [20]. Wortmannin, a PI3K family inhibitor, could effectively suppress cellular autophagy and Akt phosphorylation in the heart [11]. As such, the PI3K-dependent pathway is also a fundamental regulator that targets mitochondrial permeability transition pore (mPTP) repression by phosphorylating the transmembrane pore protein, voltage-dependent anion channel (VDAC) [27, 28]. The isoform VDAC1 is another proapoptotic protein that mediates Cyt-c release [29]. It is thus possible that wortmannin induces VDAC1 dephosphorylation, although experimental evidence is limited. In the present study, the effect of wortmannin was investigated to deepen our insights on the role of mitochondrial protection in EEP-induced cardioprotection.

## 2. Materials and Methods

### 2.1. Experimental Animals

Healthy eight-week-old male Sprague-Dawley rats (*n* = 120 rats, Siper BK, Shanghai, China) with average weights of about 252 ± 11 g were housed in cages (five rats per cage). The rats were fed with standard rodent mash and water ad libitum and maintained at constant temperature, humidity, and 12 h light/dark cycle. All animal procedures were in accordance with the institutional guidelines and approved by the Institutional Animal Care and Use Committee (NIH Publication No. 85-23, revised 1996) and approved by the Ethics Committee for Science Research of the Shanghai University of Sport.

### 2.2. Experimental Protocol

All animals were subjected to five days of adaptive treadmill running (10–20 min at 15 m/min, 0% grade), which was followed by one day of rest. On the seventh day, the rats were randomly distributed into six groups (*n* = 20 rats per group) and underwent insertion procedures that are described in the following sections. All exercise groups underwent prior warm-up at an initial velocity of 15 m/min, 0% grade for 5 min, and a 5 min symmetrical velocity increase up to 30 m/min. The exercise preconditioning protocol was as previously described [3, 30, 31]. The exhaustive exercise group (EE group) was subjected to consecutive running at 25–35 m/min, with velocities based on the acceptable maximum intensities of individual differences. The rats were allowed to run until exhaustion, which was based on the observation that the animal is unable to upright itself when placed on its back, and then sacrificed at 30 min after reaching exhaustion. The early exercise preconditioning group (EEP group) consisted of a warm-up session and an EP scenario (approximately 75% VO_2max_) [32]. The EP protocol consisted of four periods of 10 min running at 28–30 m/min with 10 min of intervallic recovery and terminated gradually via a 10 min cooldown after the last 10 min of running. The animals of the EEP group were sacrificed at 30 min after the exercise. The early exercise preconditioning plus exhaustive exercise group (EEP + EE group) included an exhaustive exercise period 30 min after the EP period and sacrificed 30 min after exhaustion. The wortmannin plus exercise preconditioning group (W + EEP group) received an intraperitoneal injection of wortmannin (Cell Signaling, MA, USA) 30 min before the EP period, using a dose of 0.7 mg/kg BW, 86 mg/mL wortmannin per 1 mL DMSO, and 0.7% DMSO solution diluted in 0.85% saline, as previously described [33]. The wortmannin plus exercise preconditioning plus exhaustive exercise group (W + EEP + EE group) received an intraperitoneal injection of wortmannin 30 min prior to the EP period, followed by exhaustion approximately 30 min later.

All rats were anesthetized by intraperitoneally injecting 10% trichloroacetaldehyde monohydrate at a dose of 400 mg/kg BW. Approximately 5 mL of blood was collected from the inferior vena cava. After 0.85% saline perfusion, for myocardial detection by using Western blot, the heart was immediately excised and the tissue close to the apex of the left ventricle wall was collected, rapidly frozen in liquid nitrogen, and then stored at −80°C. For histological analysis, supplementary 4% paraformaldehyde was perfused after saline, and the heart was excised and kept in 4% paraformaldehyde for 24 h.

### 2.3. Detection of Heart-Type Fatty Acid-Binding Protein (H-FABP) in Plasma

The blood samples were centrifuged at 3000 rpm for 15 min to collect the plasma. Chemiluminescence (CL), a double-linked ELISA technique, was performed to detect plasma H-FABP levels. Alkaline phosphatase-labeled monoantibodies of H-FABP (Kangchen Biotech, Shanghai, China) were added into the plasma samples with a surfactant-included buffer. After incubation, superparamagnetic microsphere-enveloped antibodies were added. The products were measured using an Access AccuTnI+3 troponin I kit with chemiluminescent substrates Lumi-Phos^∗^ 530 on an Access 2 immunoassay system (Beckman Coulter, Brea, CA, USA) using a linear range of 0.02–100 ng/mL.

### 2.4. Histological Handling

After 24 h of fixation, the myocardial tissues were trimmed to 1 mm^3^ cubes and then washed and immersed in PBS for 24 h (0.01 M, pH = 7.4). The tissues were then dehydrated through an alcohol gradient, dipped in different melting paraffin waxes at 60°C, and then embedded. The paraffin sections were then dewaxed to water and washed with PBS thrice, followed by a wash using distilled water. For visible light microscopic observation, the sections were stained with hematoxylin, followed by 1% hydrochloric acid alcohol to differentiate the nuclei. For each experimental group, five sections were randomly selected from different samples, as well as five nonoverlapping regions from each slice at 400x magnification. Consequently, 25 regions from each group were collected. Image acquisition and processing was performed using an image analysis system.

### 2.5. Hypoxia-Ischemia Staining

Hypoxic-ischemic changes were assessed using hematoxylin basic fuchsin picric acid (HBFP) staining. With the nucleus stained by hematoxylin, the slices were then immersed in 0.1% basic fuchsin for 3 min, washed with distilled water thrice, followed by pure acetone for 5 s, stained with 0.1% picric acid for 15 s, and then washed in acetone for 5 s. Image acquisition was performed using an optical photographic microscope (Olympus, Tokyo, Japan). Integrated optical density (IOD) and positive areas were measured using Image-Pro Plus (Media Cybernetics, Silver Springs, MD, USA).

### 2.6. Transmission Electron Microscopy (TEM)

After perfusion fixation, tissues beneath the endocardial surface of the left ventricular anterior free wall at the level of the near apex were collected for the TEM analysis. Briefly, ~1 mm^3^ tissues were fixed in 4% TEM-level paraformaldehyde (Sigma, CA, USA) and postosmicated in 1% osmium tetroxide for 2 h. After osmium fixation, the tissues were dehydrated across an acetone gradient, embedded, sectioned, and then stained with uranium acetate-lead citrate. The tissues were examined under a TEM (H-7650; Hitachi, Tokyo, Japan).

### 2.7. Immunofluorescence Staining

After dewaxing in water, the sections were washed by PBS thrice, digested with a pepsin complex at room temperature, and then immersed again in PBS. Goat serum was used as blocking reagent. For double-labeled immunofluorescence staining, antibodies were mixed, added to the tissue sections, and then incubated for 24 h at 4°C. The same procedure was used for the mixture of the anti-Bnip3 rabbit antibody (anti-rat, 1 : 500, Cell Signaling, MA, USA) and anti-TOM20 mouse antibody (anti-rat, 1 : 200, Santa Cruz, CA, USA), and the mixture of anti-LC3 rabbit antibody (anti-rat, 1 : 500, Abcam, CBG, UK) and anti-COX4/1 mouse antibody (anti-rat, 1 : 500, Sigma, CA, USA), which were, respectively, added onto different tissue slices. PBS was used as negative control instead of the primary antibody.

A fluorescein-labeled secondary antibody mixture of FITC (anti-rabbit, 1 : 200, Beyotime Biotech, Jiangsu, China) and CY3 (anti-mouse, 1 : 200, Beyotime Biotech, Jiangsu, China) was used for detection. The slides were incubated in the dark at 37°C, the nuclei were stained with DAPI, and images were acquired using a laser scanning confocal microscope (Zeiss, Jena, Germany). Immunofluorescence images of LC3 (green) + COX4/1 (red) and Bnip3 (green) + TOM20 (red) were analyzed by Zen 2012 (black version) software (Zeiss, Jena, Germany). The data of each protein were acquired using the same confocal laser scanning parameters. The fluorescence intensity data were also collected. Translocational extent was defined as the colocalization coefficient percentage (Manders' colocalization coefficients (MCC)), as described elsewhere [34]. The MCC formula is presented below. In addition, the number of COX4/1-labeled mitochondria was determined using the ImageJ software (NIH, Bethesda, MD, USA).

(1) $$\begin{array}{c} M_{1}=\frac{\sum_{i}R_{i,colocal}}{\sum_{i}R_{i}}, \end{array}$$

where *R*_*i*,colocal_ = *R*_*i*_ if *G*_*i*_ > 0 and *R*_*i*,colocal_ = 0 if *G*_*i*_ = 0 and

(2) $$\begin{array}{c} M_{2}=\frac{\sum_{i}R_{i,colocal}}{\sum_{i}R_{i}}, \end{array}$$

where *G*_*i*,colocal_ = *G*_*i*_ if *R*_*i*_ > 0 and *G*_*i*,colocal_ = 0 if *R*_*i*_ = 0.

### 2.8. Mitochondrial Isolation

Mitochondrial isolation was performed as described elsewhere [17]. Frozen heart samples were thawed in an MS buffer. The tissues were then minced and homogenized in a Polytron on ice for three cycles of 15 s. The homogenates were centrifuged at 1000*g* for 10 min at 4°C, and the crude supernatants were further centrifuged at 3000*g* for 15 min at 4°C. The mitochondria-enriched pellet was designated as the heavy membrane fraction and the supernatant as the crude cytosol. The samples were stored at −80°C until use.

### 2.9. Immunoprecipitation

Immunoprecipitation (IP) was performed as described elsewhere [35]. Myocardial tissues were homogenized in RIPA buffer, and the protease inhibitor PMSF was added. The homogenates were centrifuged at 12,000 rpm for 20 min at 4°C, and 200 *μ*L of the supernatant liquor was collected. Following the standard of 1 *μ*g antibody per 500 *μ*g protein, Tom20 antibody (Santa Cruz Biotechnology) was added for overnight incubation, and the negative control used IgG to replace the TOM20 antibody. Then, the homogenates were incubated with 40 *μ*L of Protein A/G beads (Beyotime Biotech, Jiangsu, China) for 3 h at 4°C. Beads were washed five times in RIPA buffer then centrifuged at 2500 rpm for 5 min each time at 4°C, and the immunoprecipitated proteins were used to analyze the binding relationships between Bnip3 and TOM20 by the Western blotting method. Bnip3 expression of the TOM20-immunoprecipitated products was detected and was balanced by TOM20 blotting results.

### 2.10. Western Blotting Analysis

The myocardial tissues were homogenized to directly collect the supernatant liquor or to further collect the separated mitochondrial (mito) and cytoplasmic (cyto) extracts. The phosphatase inhibitor PhosSTOP™ (Roche, Mannheim, Germany) and PMSF were freshly added. The acrylamide concentrations in the SDS gels were 10% or 15% and used to separate the proteins. The protein bands were then transferred onto PVDF membranes at 4°C. The transfer membrane was incubated in a 5% BSA solution at room temperature for 1 h to prevent nonspecific binding. After blocking, Bnip3 (Cell Signaling, USA), TOM20 (Santa Cruz, USA), LC3 (Abcam, UK), Beclin1 (Cell Signaling, USA), Bcl-2 (Santa Cruz, USA), p62 (Novus, CO, USA), Cyt-c (Santa Cruz, USA), Drp-1 (Cell Signaling, USA), p-Akt (Cell Signaling, USA), and MnSOD (Santa Cruz, USA) antibodies at 1 : 1000 dilution were added, whereas *β*-actin (Sigma, USA) and GAPDH (Sigma, USA) antibodies were added at a 1 : 1000 dilution. The COX4/1 (Sigma, USA) antibody was used at a 1 : 1000 dilution. These were then used to normalize loading of the mito extracts as previously reported [17]. The membrane was incubated overnight at 4°C to allow antigen-antibody binding. HRP-labeled secondary antibody at 1 : 5000 dilution was added, followed by incubation at room temperature for 1 h. An ECL™ chemiluminescent reaction mixture was added onto the membrane. Images of the membranes were directly captured using an automatic chemiluminescence system (Type 5200, Tanon, Shanghai, China). Images were then scanned and measured in terms of the gray value of specific bands by using ImageJ (NIH).

### 2.11. Mn^2+^-Phos-tag™ SDS-PAGE Analysis

Phos-tag acrylamide (NARD, Hyogo, Japan) at a concentration of 5 mM and supplemented with 10 mM MnCl_2_ was added to the SDS-PAGE gels. Prior to blotting, the gels were first soaked in a transfer buffer containing 8 mM EDTA for 15 min to remove MnCl_2_, and the duration of blotting was extended to 3 h [36]. The VDAC1 (Sigma, USA) antibody at a 1 : 1000 dilution was then added to determine the extent of VDAC1 phosphorylation via Phos-tag gel blotting.

### 2.12. Spectrophotometric Analysis

To detect H_2_O_2_, the xylenol orange- (FOX-) dependent colorimetric method, which is based on the principle of ferrous oxidation, was reported [37]. Following the instructions provided in the hydrogen peroxide assay kit (Jiancheng Bioengineering Institute, Nanjing, China), fresh myocardial tissues were homogenized in saline solution at a 1 : 9 volume ratio on ice and centrifuged at 2500 rpm for 10 min, Then, 100 *μ*L of the 10% concentration sample was used to react with 1 mL of the reagent solution at 37°C for 1 min. The respective optical density (OD) value of the targets at a wavelength of 405 nm was transformed to the relative content by calculating with blank sample (water) and standard sample (163 mmol/L H_2_O_2_) [38]. The consequent H_2_O_2_ levels (mmol/L) were then captured. MDA levels (nmol/mg prot) were detected using a method similar to that of H_2_O_2_. MnSOD activity (U/mg prot) was determined based on the total (T) SOD activity and deducting the CuZn-SOD activity. Protein concentrations (Coomassie Brilliant Blue), malondialdehyde (MDA) levels, H_2_O_2_ levels, and SOD activity data were acquired by using an ENOC™ microplate spectrophotometer (BioTek, VT, USA),

### 2.13. Statistical Analysis

Data were analyzed using SPSS 20.0 (IBM, Chicago, IL, USA). Two-way ANOVA and one-way ANOVA were used to determine significant group differences based on pairwise comparisons and to determine the major effects. Post hoc contrasts were analyzed using a Student-Newman-Keuls (SNK) test. The results were expressed as the mean ± SD, using *P* < 0.05 in determining statistical significance.

## 3. Results

### 3.1. EEP Provides Cardiac Adaptation to Exhaustive Exercise and Is Partly Attenuated by the Autophagy Inhibitor Wortmannin

**Figure 1:**
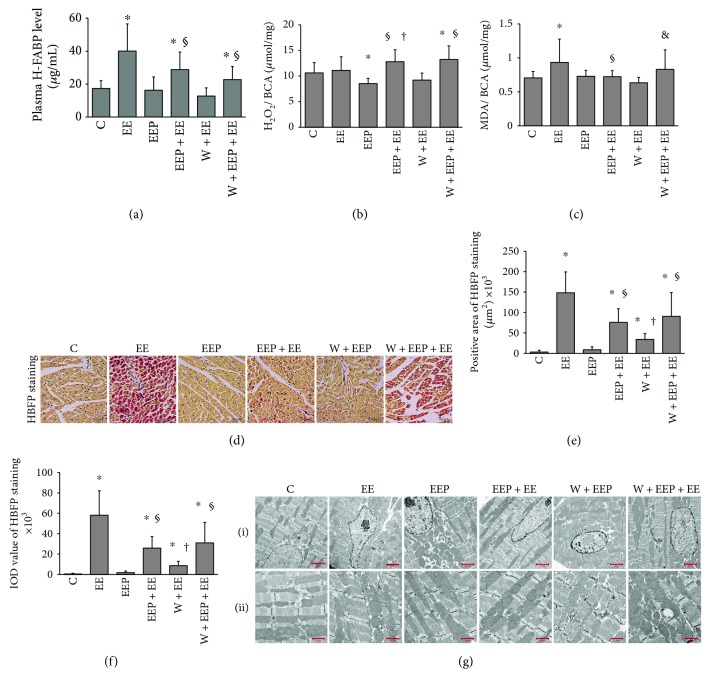
EEP provides cardioprotection against exhaustive exercise and is partly attenuated by the autophagy inhibitor wortmannin. (a) Changes in plasma H-FABP levels. Comparison of group EE to C, EEP + EE to C and EE, and W + EEP + EE to C and EEP + EE showed significant differences (*P* < 0.05). (b) Changes in H_2_O_2_ levels. Comparison of group EEP to C, group EEP + EE to EE and EEP, and group W + EEP + EE to C and EE showed significant differences (*P* < 0.05). (c) Changes in MDA levels in the myocardium. Comparison of group EE to C, EEP + EE to EE, and W + EEP + EE to EEP + EE showed significant differences (*P* < 0.05). (d) HBFP staining showed myocardial hypoxia-ischemia. The cytoplasm of nonischemic myocardial cells is stained brown. The hypoxic-ischemic area stains bright crimson. Groups EE and W + EEP + EE clearly depict hypoxia-ischemia. Original magnification was ×400, bar = 20 *μ*m. (e and f) HBFP staining and changes in integral optical density (IOD). Both data presented distinct patterns. Comparison of group EE to C, EEP + EE to C and EE, W + EEP to C and EEP, and W + EEP + EE to C and EE showed significant differences (*P* < 0.05). (g) Electron micrographs showed changes in myocardial ultrastructure in six different conditions. (g, i) Original magnification was ×1.5 K, bar = 2.0 *μ*m. Images showed morphological alterations in group EE that included karyotheca swelling, chromatin margination, myofibrillae breakage, and decrease in size and shape of the mitochondria into spheres, thereby indicating ultrastructural damages. Groups EEP and EEP + EE depict normal ultrastructural features. EEP presented numerous mitochondria that were distributed around the nucleus, which were not observed in the EEP + EE group. The groups W + EEP and W + EEP + EE exhibited EE-like morphological changes. W + EEP + EE showed excessive generation of mitochondria between myofibrillae. (g, ii) Original magnification was ×3.0 K, bar = 1.0 *μ*m. Images depict magnified ultrastructural images of the mitochondria. Groups C and EEP and EEP + EE showed normal mitochondrial morphology. Group EE was associated with mild swelling. Group W + EEP was associated with serious swelling. Group W + EEP + EE was associated with abnormally hyperplastic alterations. ^∗^Compared to C; ^§^compared to EE; ^†^compared to EEP; ^&^compared to EEP + EE. C: control; EE: exhaustive exercise; EEP: early exercise preconditioning; EEP + EE: early exercise preconditioning plus exhaustive exercise; W + EEP: wortmannin plus early exercise preconditioning; W + EEP + EE: wortmannin plus early exercise preconditioning plus exhaustive exercise.

To accurately evaluate the extent of myocardial injury induced by exercise, the plasma H-FABP levels were measured (Figure 1(a)). The EE group showed a significant increase in H-FABP levels. Using an intermittent high-intensity EP protocol for 90 min, the EEP group (0.5 h after EP) did not show a significant increase in plasma H-FABP levels. The results demonstrated that the plasma H-FABP levels in the EEP + EE group significantly decreased compared to that in the EE group. The results showed no difference between the W + EEP and EEP groups. However, no changes in plasma H-FABP levels were observed in the W + EEP + EE group compared to the EEP + EE group.

The EEP group showed a significant decrease in H_2_O_2_ levels compared to that in the C group, whereas that in the EEP + EE group was significantly higher than that in the EE and EEP groups, and that of the W + EEP + EE group was significantly higher than that of the C and EE groups (Figure 1(b)). Oxidative stress injury in cardiac tissues was indicated by marker MDA (Figure 1(c)). The EE group showed a significant increase in MDA levels. EEP + EE significantly repressed EE-induced oxidative stress injury. Wortmannin had no effect on aggravating oxidative stress injury to W + EEP. However, the MDA levels in the W + EEP + EE group were significantly higher than in the EEP + EE group, indicating that EEP-induced protection to oxidative stress injury was dampened by wortmannin.

HBFP staining showed the rat cardiomyocyte nuclei in the C and EEP groups as blue, whereas the cytoplasm was light brown, which was indicative of the absence of ischemic crimson staining (Figure 1(d)). A few red stains in the W + EEP group were observed. However, after exhaustive exercise, a significant portion of the tissues in the EE, EEP + EE, and W + EEP + EE groups stained crimson, indicating hypoxic-ischemia. Image analysis also showed a significant increase in the positively stained area (Figure 1(e)) and the IOD values (Figure 1(f)) in the EE group. A plasma H-FABP-like consistency was also detected in the EEP + EE group, which might be due to the protective effect of EEP. However, two data of HBFP staining in the W + EEP group were significantly higher than the EEP and C groups. The data in the W + EEP + EE group were significantly lower than that in the EE group. Furthermore, an increase in HBFP was observed in the W + EEP + EE group compared to that in the EEP + EE group.

The ultrastructure of the cardiomyocytes from the left ventricular anterior free wall of the C group (Figure 1(g)) apparently displayed normal morphology and was mainly characterized by smooth karyotheca, evenly distributed chromatin (Figure 1(g), (i)), well-ranged myofibrillae, and normal shape, size, and amount of mitochondria (Figure 1(g), (ii)). The EE group exhibited karyothecal swelling and chromatin marginalization and breaks in the myofibrillae. The amount of mitochondria apparently increases in number and transformed into smaller, globoid-shaped organelles. However, the mitochondria of the EE group did not show severe damage and only showed mild swelling. The morphology of the mitochondria in the EEP group was apparently normal, whereas those surrounding the nucleus exhibited fission, thereby depicting spherical shapes. However, the mitochondria that were distributed between myofibrillae were larger and elliptic or spindle in shape. The EE-induced morphological alterations were significantly suppressed by EEP + EE. In this group, the ultrastructure of the cardiomyocytes was normal, although we observed an abundance of EEP-generated mitochondria around the nuclei, which were not detected during the subsequent EE. Under the influence of wortmannin, EE-like ultrastructural damages were observed in the W + EEP group, which may be a critical factor to increasing hypoxia-ischemia. In the W + EEP + EE group, numerous hypertrophic and hyperplastic mitochondria between myofibrillae were observed compared to those in EEP + EE, and the mitochondria in the W + EEP + EE group might be associated with mitochondrial dysfunction.

### 3.2. Changes in LC3 Expression and Mitochondrial Translocation during EEP-Induced Cardioprotection

**Figure 2:**
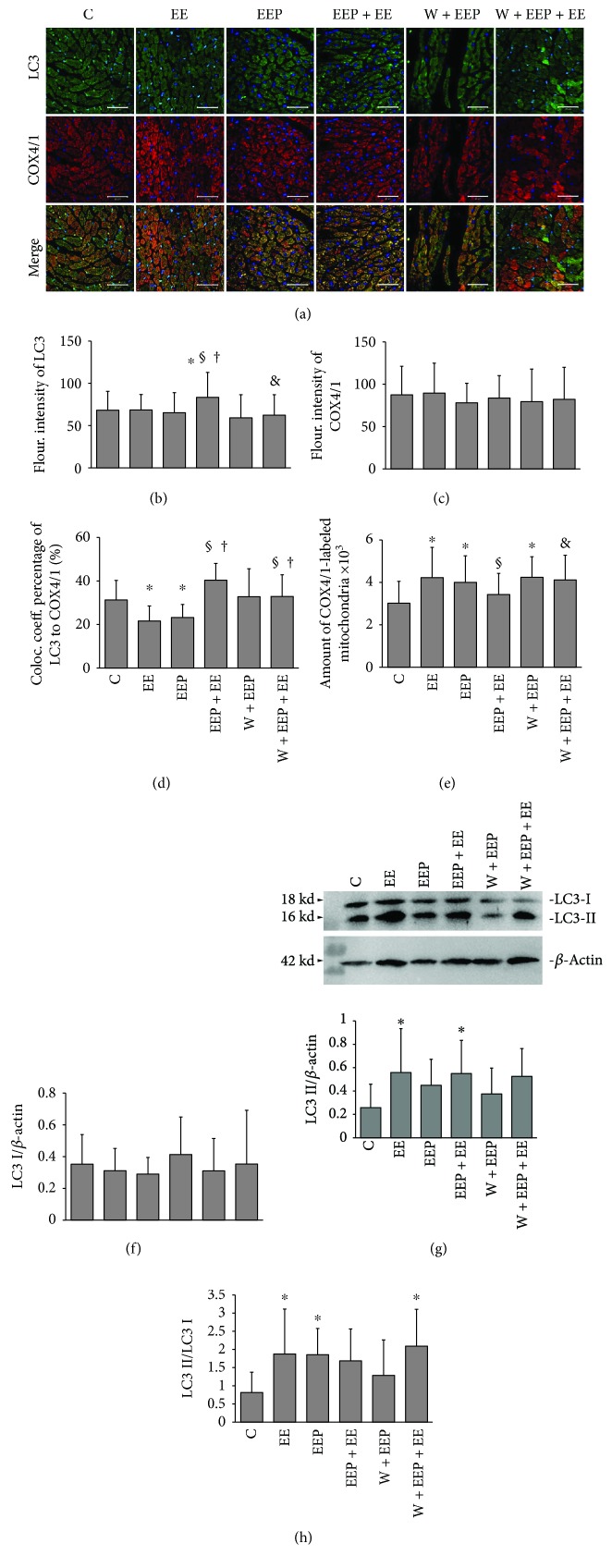
Changes in LC3 expression and mitochondrial translocation during EEP-induced cardioprotection. (a) The scanning immunofluorescence images of LC3 shows green immunoreactive products that are evenly distributed within the myocardium, which includes Pro-LC3, LC3I, and LC3II. The LC3II-dependent autophagic fluxes appeared as bright spots. The images of COX4/1 show red immunoreactive products that are distributed as granular aggregates in the myocardium of high fluorescence intensities. The merged images show a more dispersed distribution of green and red fluorescence in groups EE and EEP. Original magnification was ×400, bar = 20 *μ*m. (b) Changes in fluorescence intensity of LC3. Comparison of group EEP + EE to C, EE, and EEP, and W + EEP + EE to EEP + EE showed significant differences (*P* < 0.05). (c) No differences in fluorescence intensity of COX4/1 were observed among groups. (d) Colocalization coefficient of LC3 to COX4/1 expressed as the percentage. Comparison of groups EE and EEP to C and that of groups EEP + EE and W + EEP + EE to EE and EEP showed significant differences (*P* < 0.05). (e) Changes in the number of COX4/1-labeled mitochondria. Comparison of groups EE, EEP, and W + EEP to C, group EEP + EE to EE, and group W + EEP + EE to EEP + EE showed significant differences (*P* < 0.05). (f) Changes in immunoblotting levels of LC3I, indicating no differences among groups. (g) Changes in LC3II expression based on blotting. Comparison of groups EE and EEP + EE to C showed significant differences (*P* < 0.05). (h) Changes in the ratio of LC3II to LC3I. Comparison of groups EE, EEP, and W + EEP + EE to C showed significant differences (*P* < 0.05). ^∗^Compared to C; ^§^compared to EE; ^†^compared to EEP; ^&^compared to EEP + EE. C: control; EE: exhaustive exercise; EEP: early exercise preconditioning; EEP + EE: early exercise preconditioning plus exhaustive exercise; W + EEP: wortmannin plus early exercise preconditioning; W + EEP + EE: wortmannin plus early exercise preconditioning plus exhaustive exercise.

The scanning immunofluorescence images of LC3 showed green immunoreactive products, which represented Pro-LC3, LC3I, and LC3II, and were evenly distributed in the myocardium. The LC3II-dependent autophagic fluxes were observed as bright spots. Immunodetection of COX4/1 showed intensely stained red aggregates in the myocardium. The merged images showed an extensive distribution of green and red fluorescence in the EE and EEP groups (Figure 2(a)).

Immunofluorescence analysis indicated that the EEP + EE group underwent a significant increase in LC3 expression compared to the C, EE, and EEP groups, whereas the W + EEP + E group exhibited a significant decrease compared to the EEP + EE group (Figure 2(b)). However, no differences in fluorescence intensity were observed for COX4/1 (Figure 2(c)). The LC3 to COX4/1 colocalization coefficient percentage of the EE and EEP groups was significantly lower than that of C, and that of the EEP + EE group was significantly higher than in the EE and EEP groups. However, the LC3 to COX4/1 colocalization coefficient percentage of the W + EEP + EE group was also significantly higher than in the EE and EEP groups (Figure 2(d)). Groups EE, EEP, and W + EEP showed a significant increase in the amount of COX4/1-labeled mitochondria compared to the C group (Figure 2(e)). The EEP + EE group showed a significant decrease in the amount of COX4/1-labeled mitochondria compared to the EE group. The amount of COX4/1-labeled mitochondria in the W + EEP + EE group was significantly higher than that of EEP + EE.

Immunoblotting showed no differences in LC3I levels among various groups (Figure 2(f)). The LC3II levels of groups EE and EEP + EE significantly increased compared to the C group (Figure 2(g)). The LC3II/LC3I ratio of groups EE and EEP indicated higher degrees of autophagy (Figure 2(h)). However, despite the upregulation in LC3II, the EEP + EE group showed no change in the LC3II/LC3I ratio. However, the W + EEP + EE group exhibited a significant increase in the LC3II/LC3I ratio compared to that in the C group, in contrast to that in the EEP + EE group.

### 3.3. Changes in Bnip3 Expression and Mitochondrial Translocation during EEP-Induced Cardioprotection

**Figure 3:**
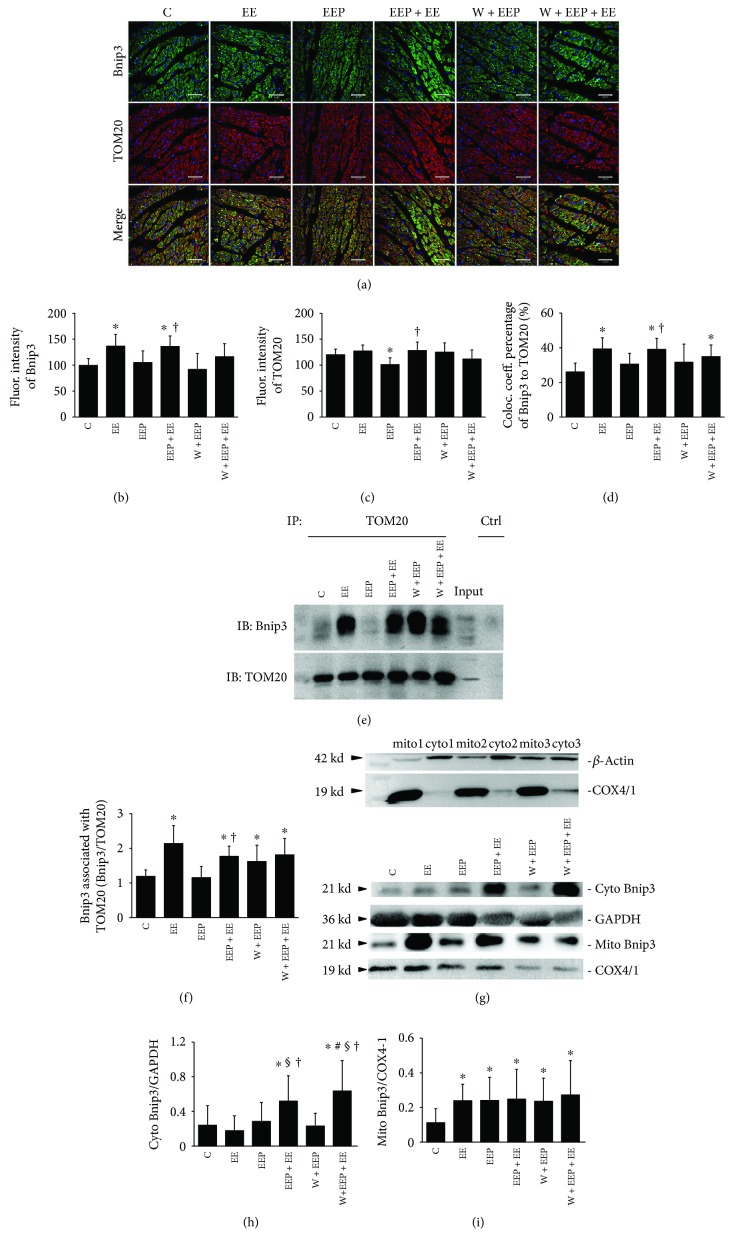
Changes in Bnip3 expression and mitochondrial translocation during EEP-induced cardioprotection. (a) Scanning immunofluorescence images of Bnip3 (green), which is dispersed across the myocardium; several cardiomyocytes in the EEP + EE group showed high-intensity fluorescence. TOM20 (red) is distributed as granular aggregates in the myocardium. The merged images show distinct differences in the distribution of red and green signals in the cardiomyocytes. Original magnification was ×400, bar = 20 *μ*m. (b) Changes in fluorescence intensity of Bnip3. Comparison of group EE to C and that of group EEP + EE to C, EE, and EEP showed significant differences (*P* < 0.05). (c) Changes in the fluorescence intensity of TOM20. Comparison of groups EEP to C, groups EEP + EE and W + EEP to EEP, and group W + EEP + EE to EE, EEP + EE, and W + EEP showed significant differences (*P* < 0.05). (d) Colocalization coefficient of Bnip3 to TOM20 as expressed as a percentage. Comparison of groups EE to C, EEP + EE to C and EEP, and W + EEP + EE to EEP showed significant differences (*P* < 0.05). (e) Coimmunoprecipitation experiment between Bnip3 and TOM20. IP: immunoprecipitated proteins; IB: immunoblotting proteins; Input: original expressions to Bnip3 and TOM20 in the nonimmunoprecipitated sample; Ctrl: negative control using IgG to replace TOM20 antibody. (f) Changes in the expression of Bnip3 blotting by TOM20 immunoprecipitation. Comparison of groups EE, EEP + EE, W + EEP, and W + EEP + EE to C and that of group EEP + EE to EEP showed significant differences (*P* < 0.05). (g) Test of mitochondrial isolation was performed using COX4/1 and *β*-action blotting in separated mitochondrial and cytosolic fractions from three independent cardiac samples, thereby indicating that the isolation was successful. (h) Changes in the expression of cytosolic Bnip3 by using immunoblotting. Comparison of group EEP + EE to C, EE, and EEP and that of group W + EEP + EE to C, EE, EEP, and W + EEP showed significant differences (*P* < 0.05). (i) Changes in the expression of mitochondrial Bnip3 by immunoblotting, relative to that of COX4/1. Groups EE, EEP, EEP + EE, W + EEP, and W + EEP + EE were significantly different (*P* < 0.05). ^∗^Compared to C; ^§^compared to EE; ^†^compared to EEP; ^&^compared to EEP + EE; ^#^compared to W + EEP. C: control; EE: exhaustive exercise; EEP: early exercise preconditioning; EEP + EE: early exercise preconditioning plus exhaustive exercise; W + EEP: wortmannin plus early exercise preconditioning; W + EEP + EE: wortmannin plus early exercise preconditioning plus exhaustive exercise.

Bnip3 was represented by green immunoreactive products that were distributed within the myocardium, and several cardiomyocytes in the EEP + EE group exhibited intense fluorescence signals. TOM20 was indicated by red immunoreactive products that were distributed as granular aggregates in the myocardium. The merged images showed differences in the distribution of red and green fluorescence in the cardiomyocytes (Figure 3(a)). Western blotting demonstrated COX4/1 expression in the mito samples, but not in almost the cyto samples (Figure 3(g)), thereby indicating that COX4/1 is suitable to label the mitochondria for the confocal imaging experiment.

The EE group showed a significant increase in Bnip3 expression compared to group C; the EEP + EE group exhibited a significant increase when compared to the C and EEP groups (Figure 3(b)). TOM20 expression in the EEP group was significantly lower than that in the C, EEP + EE, and W + EEP groups (Figure 3(c)). The EE group showed a significantly higher colocalization coefficient percentage of Bnip3 to TOM20 than the C group did (EE versus C, 39.32 ± 6.49 versus 26.04 ± 5.08, *P* < 0.05), and that of the EEP + EE group was significantly higher than those of the C and EEP groups (Figure 3(d)). The W + EEP + EE group also showed a significantly higher colocalization coefficient percentage of Bnip3 to TOM20 than the C group did, whereas no difference to that in the EEP + EE was detected.

The results of Bnip3 and TOM20 coimmunoprecipitation and Bnip3 blotting coincided with the confocal data on the colocalization coefficient percentage of Bnip3 to TOM20 (Figure 3(e)). The analysis of Bnip3 expression in immunoprecipitated TOM20 showed that groups EE, EEP + EE, W + EEP, and W + EEP + EE showed significant increases in Bnip3 expression than group C did, and that of the EEP + EE group was also significantly higher than that of the EEP group (Figure 3(f)). The cytosolic Bnip3 levels significantly increased in the EEP + EE group (EEP + EE versus C, EE, and EEP, 0.52 ± 0.29 versus 0.24 ± 0.23, 0.18 ± 0.17, and 0.28 ± 0.22, *P* < 0.05), as well as in the W + EEP + EE group compared to the C, EE, EEP, and W + EEP groups (Figure 3(h)). Relative to the mito COX4/1, the Bnip3 levels in the mito also exhibited similar increases in the EE, EEP, EEP + EE, W + EEP, and W + EEP + EE groups (Figure 3(i)).

### 3.4. Regulation of Mitochondrial Protection during EEP-Induced Cardioprotection

**Figure 4:**
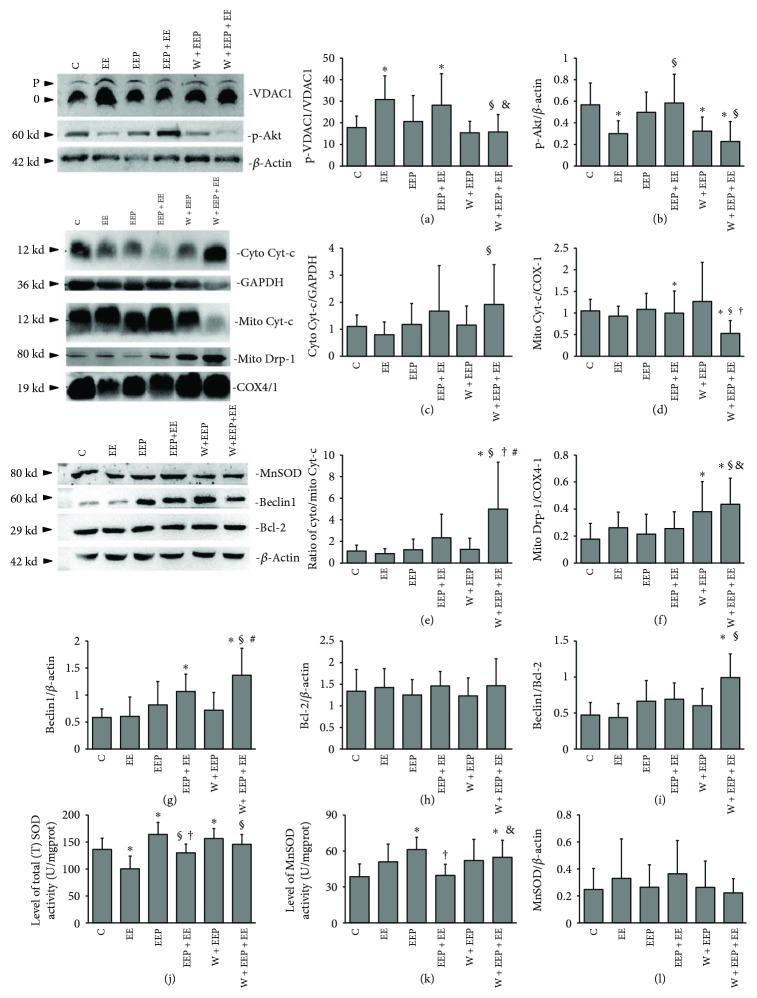
Regulation of mitochondrial protection during EEP-induced cardioprotection. (a) Changes in the ratio of phosphorylated VDAC1 (p-VDAC1) to nonphosphorylated VDAC1 (0-VDAC1), as indicated by Phos-tag blotting. Comparison of groups EE and EEP + EE to C and that of group W + EEP + EE to EE and EEP + EE showed significant differences (*P* < 0.05). (b) Changes in p-Akt expression. Comparison of groups EE, W + EEP, and W + EEP + EE to C showed significant differences (*P* < 0.05). Comparison of group EEP + EE to EE and W + EEP + EE showed significant differences (*P* < 0.05). (c) Changes in the expression of cytosolic Cyt-c by immunoblotting. Comparison of group W + EEP + EE to EE showed a significant difference (*P* < 0.05). (d) Mitochondrial Cyt-c. Comparison of group W + EEP + EE to C, EE, and EEP showed significant differences (*P* < 0.05). (e) Changes in the ratio of cytosolic Cyt-c to mitochondrial Cyt-c. Comparison of group W + EEP + EE to EE showed a significant difference (*P* < 0.05). (f) Changes in mitochondrial Drp-1 expression. Comparison of group W + EEP to C and that of group W + EEP + EE to C, EE, and EEP + EE showed significant differences (*P* < 0.05). (g) Changes in Beclin1 expression. Comparison of group EEP + EE to C and that of group W + EEP + EE to C, EE, and W + EEP showed significant differences (*P* < 0.05). (h) Bcl-2 expression did not differ among groups. (i) Changes in the ratio of Beclin1 to Bcl-2. Comparison of group W + EEP + EE to C showed a significant difference (*P* < 0.05). (j) Changes in total (T) SOD activity. Comparison of groups EE, EEP, and W + EEP to C, group EEP + EE to EE and EEP, and group W + EEP + EE to EE showed significant differences (*P* < 0.05). (k) Changes in MnSOD activity. Comparison of groups EEP and W + EEP to C, group EEP + EE to EEP, and group W + EEP + EE to C and EE showed significant differences (*P* < 0.05). (l) Measurement of MnSOD expression levels by immunoblotting showed no differences among groups. ^∗^Compared to C; ^§^compared to EE; ^†^compared to EEP; ^&^compared to EEP + EE; ^#^compared to W + EEP. C: control; EE: exhaustive exercise; EEP: early exercise preconditioning; EEP + EE: early exercise preconditioning plus exhaustive exercise; W + EEP: wortmannin plus early exercise preconditioning; W + EEP + EE: wortmannin plus early exercise preconditioning plus exhaustive exercise.

Phos-tag blotting indicated that the ratio of the phosphorylated VDAC1 (p-VDAC1) to the nonphosphorylated VDAC1 (0-VDAC1) increased in the EE and EEP + EE groups, but was reduced by W in the W + EEP + EE group (Figure 4(a)). The phosphorylated Akt (p-Akt) levels decreased in the EE, W + EEP, and W + EEP + EE groups compared to those of the C group (Figure 4(b)), whereas these increased in the EEP + EE group relative to those in the EE and W + EEP + EE groups. The cyto Cyt-c levels in the W + EEP + EE group were significantly higher than those in the EE group (Figure 4(c)). The relative mito Cyt-c levels in the W + EEP + EE group were significantly lower than those of the C, EE, and EEP + EE groups (Figure 4(d)), whereas the cyto Cyt-c/mito Cyt-c ratio increased with Cyt-c leakage in the W + EEP + EE group (Figures 4(e)). The mito Drp-1 levels increased in the W + EEP group compared to those in the C group (Figure 4(f)), as well as in the W + EEP + EE group relative to those in groups C, EE, and EEP + EE.

The EEP + EE groups showed a significant increase in Beclin1 expression in the entire tissue compared to C, and similar findings were observed in the W + EEP + EE group (Figure 4(g)). Bcl-2 levels did not differ among various groups (Figure 4(h)). However, a significant increase in the Beclin1/Bcl-2 ratio was detected in the W + EEP + EE group only (Figure 4(i)). The EE group showed a significant decrease in total (T) SOD activity levels, whereas those of the EEP and W + EEP groups exhibited a significant increase compared to the C group. In addition, the EEP + EE group showed a decrease in total (T) SOD activity levels relative to that of the EEP group, whereas an increase was observed compared to that in the EE group. In addition, the total (T) SOD activity levels in the W + EEP + EE group were significantly higher than those of the EE group (Figure 4(j)). Groups EEP and W + EEP showed a significant increase in MnSOD activity levels compared to that in the C group, whereas that of the EEP + EE group was significantly lower than that of the EEP group (Figure 4(k)), and that of the W + EEP + EE group was significantly higher than those of the C and EEP + EE groups. However, comparison among all groups indicated that the MnSOD blotting levels did not change (Figure 4(l)).

## 4. Discussion

### 4.1. A Low Level of Mitochondrial Protection Prevents Extensive Damage during Exhaustive Exercise

Heart-type fatty acid-binding protein (H-FABP) has recently been utilized as a clinical indicator of ischemic cardiomyopathy, as well as an animal model for I/R injury [39, 40]. In contrast, because of the influences of skeletal muscle *in vivo*, CK and LDH lack heart specificity in peripheral blood. Exhaustive exercise has been reported to result in abnormal cardiac reactions such as decreased stroke volume, ejection fraction, and total peripheral resistance in rat, possibly due to an increase in heart rate [8, 41]. Similar alterations in heart function have also been observed during the ischemic phase of an I/R injury animal model [42]. Furthermore, acute exercise-induced cardiac injury has been strongly associated with cardiac H-FABP leakage [43]. In our modeling studies, we have observed that cardiac injury-related biomarkers such as H-FABP can be stably employed in assessing exhaustion-induced myocardial injury [3, 31, 44].

Continuous high-intensity exercise until exhaustion results in abnormal myocardial alterations, as well as mitochondrial dysfunction [45]. In the present study, the plasma H-FABP levels increased in the EE group, which coincided with ultrastructural damages, particularly breakage in myofibrillae. A previous I/R injury study showed that myofibril damage may be due to oxidative stress that triggers sarcolemma destabilization [46]. Membrane lipid hydroperoxides such as MDA are generated when the membrane of cardiomyocytes is subjected to oxidant stress, which is also associated with an acute ischemia-stimulated contractile improvement [47]. We observed an increase in MDA levels in the EE group, demonstrating an increase in the risk for oxidative stress injury. Exhaustive exercise involves basic affinity mechanisms that are related to acute ischemic stress [48]. In the present study, HBFP staining indicated that exhaustive exercise is associated with hypoxic-ischemic aggravation. However, exercise-induced acute redox unbalance has been also known to function as a key signaling pathway that provides cardioprotection [49, 50]. We did not observe H_2_O_2_ induction in the EE group, which might be related to the suppression in mitochondrial MnSOD and cellular T-SOD activities. EE-induced increases in myocardial MDA levels are potentially governed by other types of ROS, particularly superoxide anions [14].

No distinct ultrastructural damages to the mitochondria were observed in this study, although these organelles were smaller and spherical in shape. Additionally, as indicated by the COX4/1-labeled mitochondrial data, results demonstrate that EE is strongly associated with mitochondrial fission [51]. Drp-1 plays a key role in mitochondrial fission; however, the findings of the present study do not support the notion of mitochondrial Drp-1 upregulation during EE. Drp-1 activation has been reported to rely on PKC*ε*-mediated phosphorylation [52]. Furthermore, we recently described the association between EE and elevation of PKC*ε* levels [3]. Thus, mitochondrial fission during EE presumably depends on Drp-1 phosphorylation, but not on Drp-1 content in the mitochondria. The observed fissions in the EE reflect potentially mitochondrial dysfunction that leads to mPTP opening, followed by the release of mitochondrial Cyt-c into the cytosol [53]. However, EE-induced Cyt-c leakage was not observed in the present study, therefore indicating that another endogenous mechanism may be blocking the connections between mitochondrial dysfunction and cellular death in EE.

VDAC1 is a VDAC isoform that consists of 19*β*barrel proteins and is responsible for the transfer of apoptotic Ca^2+^ signals from the endoplasmic reticulum (ER) and into the mitochondria [54]. VDAC1 is activated by voltage, pH, intracellular calcium concentrations, and oxidation [54]. EE-induced oxidation highly likely triggers VDAC1 activation. However, VDAC1 activation induces a feedback mechanism for the inhibition of VDAC1 [27]. In the EE group, VDAC1 may fail to mediate mPTP opening because of PI3K- and PKC*ε*-modulated VDAC1 phosphorylation [27, 28, 55]. We have previously demonstrated that myocardial PKC*ε* is upregulated in EE [3]. PKC*ε* directly binds to VDAC1 in cardiac myocytes [55]. We found that EE induces a decrease in Akt phosphorylation, indicating suppression in the PI3K pathway. The increase in VDAC1 phosphorylation in EE is presumably triggered by the PKC*ε* pathway, which in turn reduces Cyt-c release [55]. The ROS signal-dependent PKC*ε* activation may thus not be related to H_2_O_2_ in EE [16]. In addition, proapoptotic VDAC1 is an isoform that interacts with the translocalized BH-3 protein Bax that enlarges channels and prolongs mPTP opening [29]. Thus, because Bnip3 is an OMM-translocational inducer of Bax that forms aggregates in the mitochondria during EE, VDAC1-Bax channels should have been formed [8, 56]. However, the upregulated VDAC1 phosphorylation levels prevent the EE-induced mitochondrial dysfunction.

Mitophagy plays an essential protective role in acute cardiovascular stress [17, 57]. However, autophagosome proteins such as LC3 do not contribute to the mitochondrial accumulation during EE, and although an increase in the LC3II to LC3II/LC3I ratio was observed, LC3II accumulation might have likely resulted from an increase in the LC3II/I ratio during acute stress [58, 59]. Similarly, an increase in Beclin1, which is an autophagic inducer, has not been observed in EE [60, 61]. These results further indicate that EE does not induce cellular macroautophagy in the proper sense. By inhibiting the opening of the mPTP channel, phosphorylated VDAC1 may preserve the *ΔΨ*m [62]. Interestingly, as confirmed by colocalization and immunoprecipitation experiments, increased levels of OMM-translocalized Bnip3 did not contribute to LC3 recruitment during EE, and its underlying mechanism remains unclear. In conclusion, EE failed to induce mitophagy repair for mitochondrial degradation, but mPTP suppression in EE via VDAC1 phosphorylation provides limited mitochondrial protection.

### 4.2. Increased H_2_O_2_ Signaling Levels Enhance Mitochondrial Protection during EEP

H_2_O_2_ signals are essential inducers of IP-induced cardioprotection [63]. H_2_O_2_ preconditioning also assists in cardioprotection through a PI3K-dependent pathway [64]. By inhibiting PI3K by LY294002, H_2_O_2_ treatment reversely induces apoptosis of cardiomyocytes [65]. EEP + EE induced an increase in H_2_O_2_ levels, possibly providing cardioprotection. EEP + EE significantly suppressed the activity of EE-induced cardiac myofibrillae, hypoxic-ischemia, and the occurrence of ultrastructural injuries. During H_2_O_2_ upregulation, no evidence of MDA-mediated aggravation of lipid peroxidation was observed in the EEP + EE group. H_2_O_2_-independent oxidative stress injury induced by EE was mainly repressed in the EEP + EE group. A lower level of EP-induced H_2_O_2_ production provided cardioprotection to suppress subsequent acute oxidative stress [66]. Likewise, our study indicated that EEP is strongly associated with an increase in T-SOD and MnSOD activity. A previous study showed that an increase in SOD activity contributes to the downregulation of H_2_O_2_, which could then act as an adaptive mechanism to subsequent EE-dependent cardiac dysfunction and excessive oxidation [43]. Therefore, the excessive production of ROS during EE will be scavenged by the enhanced SOD activities of EEP, thereby further forming H_2_O_2_ during EEP + EE. A previous study has shown that the peak time of MnSOD mRNA synthesis occurs at 96 h after intermittent exercise, and this may explain why the expression of MnSOD did not change during EEP [49, 67]. Furthermore, wortmannin had no effect on EEP in reversing various impairments, but the degree of hypoxia-ischemia showed a small increase. These observations were possibly due to the inability of wortmannin to suppress the elevation of EEP-induced SOD activities. Wortmannin effectively abolished EEP-induced protection against oxidative stress, and the excessive production of H_2_O_2_ in the W + EEP + EE group was triggered by W + EEP-induced SOD activation, which in turn caused MDA accumulation that was mainly due to the PI3K pathway and/or autophagy inhibition.

Cardiac mitochondrial fission triggered by oxidation induced Ca^2+^ translocation through mitochondria-ER interactions [68]. COX4/1 showed that EEP is involved in the increase in the number of mitochondria. Similarly, the mitochondria surrounding the nucleus showed a spherical shape. Thus, we hypothesized that EEP upregulated protein synthesis in the ER, potentially inducing the mitochondria surrounding the nucleus to undergo fission [3, 30, 68–70]. These mitochondria were degraded during the subsequent EE. EEP + EE was also coupled with a decrease in the number of mitochondria, as indicated by the COX4/1 data. Mitochondrial fission in EEP may thus be an adaptation to subsequent acute EE. Furthermore, we observed that EEP and EEP + EE do not lead to ultrastructural impairments. The W + EEP group showed an increase in hypoxia-ischemia during the subsequent EEP. Furthermore, the increase in mitochondrial dysfunction was observed in response to wortmannin treatment in both the W + EEP and W + EEP + EE groups. The W + EEP + EE group exhibited swelling, which was validated by an increase in the number of COX4/1-labeled mitochondria. Taken together, we demonstrated that the W + EEP + EE group had insufficient mitochondrial clearance compared to EEP + EE, which was possibly associated with wortmannin-induced inhibition of autophagy resulting in increased oxidative injury [11]. Therefore, EEP-induced cardioprotection does not affect the morphology of mitochondria, and mitophagy plays a role in protecting against EE-induced mitochondrial alterations.

Aggravated Cyt-c leakage was only observed in the W + EEP + EE group; excessive H_2_O_2_ production in the W + EEP group reversed the mitochondrial damages and cellular apoptosis during W + EEP + EE [65]. These findings indicate that the PI3K pathway can be inhibited by wortmannin for EEP cardioprotection. VDAC1 and Akt were significantly phosphorylated by EEP + EE, which then were attenuated by wortmannin pretreatment in W + EEP + EE. Thus, PI3K plays a key regulator in moderating mPTP opening in EEP + EE, but not H_2_O_2_-independent PKC*ε* activation [3, 27, 28]. Following the downregulation of VDAC1 phosphorylation, the elevated H_2_O_2_ signals in W + EEP further led to Cyt-c leakage in W + EEP + EE. The Cyt-c released from the IMM induces electron leakage, which in turn increases the oxidative stress levels [71]. The normal contractile function of the heart mainly relies on ATP, which is provided by the mitochondria [72]. Improved and irreversible mitochondrial dysfunction in W + EEP + EE may reduce cardiac adaptations during EE.

Increases in Beclin1 levels, LC3 intensity, and LC3II levels contributed to the H_2_O_2_- and PI3K-dependent induction of cardioprotection during EEP + EE. EEP induced an increase in LC3II/LC3I ratio, whereas no change in LC3II was observed, thereby suggesting that EEP itself induces limited autophagy activation by transforming intrinsic LC3, and EEP does not lead to an EE-like accumulation of autophagosomes. However, such LC3 changes in EEP were independent of H_2_O_2_ and might have been triggered by other autophagic factors [73]. Thus, although LC3I changes were not significant, the increase in the LC3II/LC3I ratio and unchanged LC3 intensity indicate the potential consumption of LC3. These alterations in EEP may play a role in the endogenic stimulation of subsequent EE. The present study also showed that EE leads to increases in autophagy. Therefore, additional LC3 consumption could increase the concentration of ULK1, then through the phosphorylation induced by ULK1 activate Beclin1 to generate more LC3II during EEP + EE [74]. As a consequence of EEP + EE, the increase in the total LC3 levels was possibly triggered by PI3K-induced LC3 mRNA synthesis and by high levels of Beclin1, which surpassed LC3 consumption which was positive to the protection [75]. The autophagy inhibitor wortmannin significantly suppressed various changes in LC3 that were induced by EEP and EEP + EE, besides a high LC3II/LC3I ratio, thereby indicating autophagic obstruction during W + EEP + EE. Wortmannin had no effect on reducing Beclin1 expression and in turn induced an increase in the Beclin1/Bcl-2 ratio, which was associated with the upregulation of cytosolic Bnip3 levels [76]. Beclin1 is also induced by ROS-dependent NF-*κ*B modulation [22]. However, wortmannin pretreatment led to the production of BH-3 proteins such as Beclin1 and Bnip3, which are incapable of inhibiting presurvival Bcl-2, which then results in autophagic death, and are also associated with the H_2_O_2_ and the Cyt-c releasing predominant programmed apoptosis [77, 78].

The findings of the present study indicate that H_2_O_2_ may play a key role in activating LC3 translocation. The decrease in H_2_O_2_ levels negatively affected Bnip3 translocation in EEP, thereby further reducing LC3 accumulation in the mitochondria. In addition, the unchanged Bnip3 translocation and coimmunoprecipitation with TOM20 in the EEP, as well as its strong association with decreased levels of TOM20, may be due to an increase in the number of mitochondria that undergo fission [79, 80]. The increase in H_2_O_2_ production contributes to mitophagy, which is repressed by the overexpression of MnSOD activation [81]. Thus, increased Bnip3-mediated mitophagy is dependent on intracellular H_2_O_2_ signals in EEP + EE. The increase in VDAC1 phosphorylation that was induced by the H_2_O_2_-dependent PI3K pathway inhibited OMM depolarization. Lee et al. [24] reported that LC3 translocation is the consequence of Bnip3 activity. Bnip3 had a high extent to OMM translocation which presumably contributed to LC3 OMM accumulation in EEP + EE. Because total LC3 and LC3II were suppressed by wortmannin treatment, the elevated concentrations of mitochondria-localized Bnip3 were insufficient for downstream reactions, thereby failing in inducing mitophagy. However, OMM-localized Bnip3 during W + EEP + EE further resulted in mitochondrial damages.

## 5. Conclusions

Exhaustive exercise is accompanied by negative changes in cardiac myofibrillae, hypoxia-ischemia, and oxidative stress. However, exhaustion is associated with a limited mitochondrial protection that involves mitochondrial fission and H_2_O_2_-independent oxidative manner. The details on the mechanism on oxidation-assisted protection during EE remain unclear. EEP significantly reinforced oxidation-protection and suppressed injuries to EE by enhancing Beclin1-dependent autophagy and Bnip3-dependent mitophagy due to an adaptive promotion. EEP-induced cardioprotection is strongly associated with H_2_O_2_ signaling, in which additional PI3K and PI3K-dependent autophagy inhibitions in turn cause the H_2_O_2_-induced proapoptosis and oxidative injury.

## Data Availability

The data used to support the findings of this study are available from the corresponding author upon request.
